# Maternal mortality following thromboembolism; incidences and prophylaxis strategies

**DOI:** 10.1186/s12959-020-00251-w

**Published:** 2020-11-30

**Authors:** Mahboobeh Shirazi, Behrokh Sahebdel, Mahnoosh Torkzaban, Elham Feizabad, Marjan Ghaemi

**Affiliations:** 1 Maternal, Fetal and Neonatal Research Center, Tehran University of Medical Sciences, Tehran, Iran; 2 Yas Hospital, Tehran University of Medical Sciences, Tehran, Iran; 3 Department of Radiology, Thomas Jefferson University, Philadelphia, PA USA; 4 Valiasr Reproductive Health Research Center, Tehran University of Medical Sciences, Tehran, Iran

**Keywords:** Maternal mortality, Pregnancy, Thromboembolism, Heparin

## Abstract

**Background:**

Thromboembolism is one of the main causes of maternal mortality, which can be prevented in many cases. The present study was designed to investigate the incidence and prophylaxis strategies for maternal mortality following thromboembolism in postnatal.

**Methods:**

In this case series study, the data of the mortality cases were extracted according to the ethical and security standards of the Ministry of Health of the country and compared with a healthy control group. The thromboembolism risk factors measured and scored using a questionnaire entitled “the evaluation of risk factors for maternal mortality following thromboembolism during pregnancy, labor, or post-partum”.

**Results:**

The maternal mortality rate was 16 per 100,000 live births. Among 297 mortality cases, 27 (9%) death were due to thromboembolism. The mean gestational age was 32.5 weeks. Dyspnea (88.8%) and tachycardia (18.5%) were found as common clinical manifestations in these patients. Sixteen cases (59.3%) did not get heparin, 6 (22.2%) received single dose and 5 (18.5%) received two doses and more. In these 11 cases, 5 (45%) patients received heparin before surgery, 1 after surgery, and 5 before and after surgery. Twenty cases deceased in the first hours after delivery and the rest after 2 to 12 days. The average score of risk for thromboembolism based on Royal College of Obstetricians & Gynecologist (RCOG) guideline was 4.6.

**Conclusion:**

It seems that one of the most important cause of maternal mortality in this study was the lack of recognition of high-risk patients and the lack of prescription for prophylaxis with heparin and this clearly explains the need for accurate screening of high-risk mothers, designing a standard form and the care and treatment of these patients.

## Introduction

Maternal mortality is one of the greatest tragedies with wide ranging consequences for the family and society [1]. It is an important public health indicator that reflects both the quality of health care services and the women’s status [2, 3]. Reduction of maternal mortality serves as one of the most important international development goals [4]. Several factors such as gravidity, type of delivery, socioeconomic status was found as the most important determinants of maternal mortalities in the region [3].

Primary causes of maternal mortality in U. S include infection, cardiac disease, hemorrhage, thromboembolism and hypertensive disorders [5]. Iran has also experienced a transition pattern in its major causes of maternal mortality and morbidity from those characteristics of developing nations such as hemorrhage and infection to causes more commonly seen in developed nations including thromboembolism, cardiac diseases [6–8] and in not rare condition, post-operative complications [9]. Accurate recognition and attention on maternal mortality is critical to have an effective prevention [10].

Pregnancy is a prothrombotic and temporary condition that increase the risk of venous thromboembolism (VTE) four to five times as compared to non-pregnant women [11, 12]. The risk of venous thromboembolism is higher in women ages 35; Thrombophilia, lupus, cardiac disease, sickle cell disease, obesity, fluid and electrolyte imbalance, postpartum infection and transfusion are the other significant risk factors [11]. Many cases of VTE occur during the first trimester of pregnancy and the risk increases with progression of gestational age and reaches the maximum after delivery [13].

The incidence rate of thromboembolic events related to pregnancy is 3 people per 1000 cesarean and also suggests that cesarean delivery is associated with a fourfold increase in the risk of VTE incidence compared with vaginal delivery [14]. Women with preeclampsia are at risk of treating thromboembolic events, three times more than healthy women, in the postpartum period. There is also a correlation between smoking and the increased risk of VTE [15].

The most common signs were dyspnea at first and tachycardia in the second grade. Although these symptoms can be completely similar in other illnesses such as heart disease or bleeding, but it indicates that these symptoms should consider in pregnant women. On the other hand, these two signs seem to be the most endearing signs of the disease, and in order to prevent maternal mortality, it should be determined by accurate screening of high-risk mothers at an earlier stage. An X-ray of chest helps to exclude other causes that lead to dyspnea and tachycardia [16].

Therefore, thromboembolism risk assessment should be performed at any time during pregnancy; in early pregnancy and specially at delivery and if risk factors change, or in cases with medical or surgical conditions [17]. Identification of these factors is important and may reduce the incidence and improve its timely diagnosis. In the current study we estimate the incidence, risk factors, and mortality from pregnancy-related venous thromboembolism.

## Materials and methods

In this retrospective study cases with thromboembolism coded as the underlying or contributory cause of death in women aged 15–44 from March 2017 to February 2018 were evaluated. This national data was obtained from ministry of health which records maternal mortality statistics throughout the country, therefore no case would miss in this era. We included all women with fatal VTE occurred during pregnancy or within 6 weeks post-partum, according to the definition of maternal death. Medical records, autopsy reports and data from the Ministry of Health were examined in order to detect contributing factors for VTE.

Obstetrical information such as parity, pregnancy and delivery complications. Were retrieved either. The study protocol was approved by the local ethics committee of Tehran University of Medical Sciences. Patient records extracted in accordance with the ethical and security guidelines of the Ministry of Health and the risk factors of thromboembolism measured using a questionnaire entitled “Risk factors for thromboembolism during pregnancy and puerperium” that is summarized with the scores based on Royal College of Obstetricians & Gynecologist (RCOG) guideline [18] in Table 1. The scores will gather to reach a total value.

**Table 1:** The evaluation of risk factors for thromboembolism during pregnancy and puerperium

Risk Factors	Score
**A) Risk factors related to general conditions**	
**Body mass index (BMI) equal to or greater than 40 (before or during early pregnancy)**	2
**Age older than 35 years**	1
**BMI between 35 and 40 (before or early pregnancy / weight greater than 80 kg)**	1
**Parity ≥ 3 regardless of the current pregnancy**	1
**Smoking**	1
**Gross varicose veins (symptomatic or above the knee or with phlebitis, edema, skin changes)**	1
**B) Risk factors related to medical conditions**	
**History of previous VTE (except for the reason of major surgery)**	4
**Acquired thrombophilia (antiphospholipid antibody syndrome): at least one laboratory test and at least a clinical criterion**	4
**History of previous VTE due to major surgery**	3
**Any medical conditions: cancer, cardiac disease, active lupus, inflammatory poly-arthropathy or inflammatory bowel disease, nephrotic syndrome, type 1 diabetes mellitus with nephropathy, tuberculosis, current intravenous drug addiction**	3
**High risk inherited thrombophilia (deficiency of anti-thrombin, protein S or C deficiency, homozygote low risk thrombophilia or more than one heterozygote low risk thrombophilia)**	3
**Low risk inherited thrombophilia (heterozygote factor 5 Leiden, G20210A prothrombin gene mutation)**	1
**Family history of VTE in first degree relatives (spontaneous or estrogen dependent)**	1
**Anti-phospholipid antibodies (only laboratory criteria, without a clinical criteria)**	1
**C) Obstetrical Risk Factors**	
**Cesarean section in labor**	2
**Current preeclampsia**	1
**Pregnancy with fertility IVF / ART methods (only considered during pregnancy)**	1
**Multiple pregnancy**	1
**Non-emergency cesarean (elective)**	1
**Instrumental delivery**	1
**Prolonged labor (more than 24 h)**	1
**Postpartum hemorrhage (greater than 1 l / blood transfusion)**	1
**Preterm labor (less than 37 weeks) in the current pregnancy**	1
**Intrauterine fetal demise (IUFD) in the current pregnancy**	1
**D) Transient Risk Factors**	
**Ovarian hyper stimulation syndrome (only in the first trimester)**	4
**Surgery during pregnancy or postpartum (curettage, tubal closure, appendectomy..., except for perineal immediate repair)**	3
**Severe vomiting of pregnancy (hyperemesis)**	3
**Systemic infections (antibiotics or admission needed) foe example: pneumonia, pyelonephritis, postpartum wound infection**	1
**Hospitalization or immobilization (≥3 days rest in bed), dehydration**	1

If total score is 4 or more, anticoagulant administration with prophylaxis dose starts from the beginning of the pregnancy. In score 3, it is recommended to start anticoagulant with prophylactic dosage from the 28th week of pregnancy. If total score is 2 or more postnatally or admitted to hospital antenatally, consider thromboprophylaxis for at least 10 days. In prolonged hospitalization (≥ 3 days) or readmission to hospital within the puerperium, consider thromboprophylaxis either [18].

### Statistical analysis

Mean (SD) (standard deviation) and percentage of frequency were calculated for descriptive objectives. The data were analyzed by the SPSS version 18.

## Results

The maternal mortality rate was 16 per 100,000 live births. Among 297 mortality cases in 1 year, 27 deaths were due to thromboembolism. The mean (SD) age was 33.2 (3.3) years and the mean of gestational age was 33 weeks+ 6 days.

Two cases with mortality in the first day were not recognized and the symptoms were misdiagnosed with postpartum pain and distress. Dyspnea (88.8%) and tachycardia (18.5%) were the common manifestations in these patients. The diagnosis of all cases was confirmed by autopsy. Demographic and clinical data of the cases are listed in Table 2.

**Table 2:** Demographic and clinical data of the cases deceased due to thromboembolism

variable			N (%)
**Type of hospital**	Educational		21 (77.8)
	Private		5 (18.5)
	Charity		1 (3.7)
**BMI**^**a**^	< 30		23 (85.2)
	30–40		4 (14.8)
	≥ 40		0
**Blood Group**	A^+^		11 (40.7)
	O^+^		8 (29.6)
	B^+^		5 (18.5)
	AB^+^		2 (7.4)
	O^−^		1 (3.7)
	A^−^		0
	B^−^		0
	AB^−^		0
**Delivery Type**	NVD^b^		6 (22.2)
	CS^c^	Emergency	7 (25.9)
		Elective	14 (51.9)
**Parity**	≥3		10 (37)
	< 3		17 (63)
**Infant gender**	Male		14 (51.9)
	Female		13 (48.1)
**1st min. Apgar Score**	0		6 (22.2)
	5		1 (3.7)
	6		1 (3.7)
	7		4 (14.8)
	8		3 (11.1)
	9		12 (44.4)
**Death time**	Morning shift		18 (66.7)
	Evening shift		9 (33.3)
**Delivery to death (days)**	1		20 (74.1)
	2		3 (11.1)
	3		1 (3.7)
	4		1 (3.7)
	7		1 (3.7)
	12		1 (3.7)
**Blood transfusion**	Yes		11 (40.7)
	No		16 (59.3)
**Heparin prescription**	Yes	Single dose	6 (22.2)
		multiple doses	5 (18.5)
	No		16 (59.3)
**Time of Heparin prescription**	Before surgery		5 (18.5)
	After surgery		1 (3.7)
	Before & after surgery		5 (18.5)
	None		16 (59.3)
**Preterm labor**	Yes		13 (48.1)
	No		14 (51.9)
**IUFD**^**d**^	Yes		7 (25.9)
	No		20 (74.1)
**Postpartum hemorrhage**	Yes		7 (25.9)
	No		20 (74.1)
**Prolonged labor**	Yes		1 (3.7)
	No		26 (96.3)
**Instrumental delivery**	Yes		2 (7.4)
	No		25 (92.6)
**Sepsis**	Yes		3 (11.1)
	No		24 (88.9)
**Multiple pregnancy**	Yes		3 (11.1)
	No		24 (88.9)
**IVF/ART**^**e**^	Yes		4 (14.8)
	No		23 (85.2)
**Antiphospholipid syndrome**	Yes		1 (3.7)
	No		26 (96.3)
**Cardiac Disease**	Yes		8 (29.6)
	No		19 (70.4)
**Hypothyroidism**	Yes		1 (3.7)
	No		26 (96.3)
**Cancer**	Yes		1 (3.7)
	No		26 (96.3)
**Diabetes**	Yes		4 (14.8)
	No		23 (85.2)
**Rheumatoid disease**	Yes		1 (3.7)
	No		26 (96.3)
**Hereditary thrombophilia**	Yes		1 (3.7)
	No		26 (96.3)
**Preeclampsia**	Yes		1 (3.7)
	No		26 (96.3)
**Chronic hypertension**	Yes		3 (11.1)
	No		24 (88.9)
**Thromboembolism history**	Yes		1 (3.7)
	No		26 (96.3)

^a^body mass index, ^b^normal vaginal delivery, ^c^cesarean section, ^d^intra uterine fetal demise, ^e^in vitro fertilization/ assisted reproductive technology

There were no any case of family history for thromboembolism, hospitalized or immobilized (≥3 days rest in bed), dehydration, ovarian hyperstimulation syndrome, surgery during pregnancy or postpartum, hyperemesis gravidarum, nephropathy disorders, smoking, intravenous drug addiction, inflammatory bowel disease and sickle cell disease among these patients.

The mean (SD) of thromboembolism score was 4.6 (1.9). Nine patients had score < 4 and 18 patients ≥4. Heparin prescription frequencies based on different reasons are listed in Table 3.

**Table 3:** Heparin prescription frequencies based on different reasons

variable		Heparin**+**	Heparin**−**
		N (%)	N (%)
**Total score < 4**		2 (22.2)	7 (77.8)
**Total score ≥ 4**		9 (50.0)	9 (50.0)
**Death in morning shift**		2 (22.2.0	7 (77.8)
**Death in night shift**		9 (50.0)	9 (50.0)
**Normal Vaginal Delivery**		3 (50.0)	3 (50.0)
**Cesarean Section**		8 (38.1)	13 (61.9)
**Body Mass Index < 30**		11 (40.7)	12 (59.3)
**Body Mass Index ≥ 30**		0	4 (100)
**Parity ≥ 3**		4 (40.0)	6 (60.0)
**Parity < 3**		7 (42.2)	10 (58.8)
**Cardiac Disease**	Yes	4 (50.0)	4 (50.0)
	No	7 (36.8)	12 (63.2)
**Preterm Labor**	Yes	6 (46.2)	7 (53.8)
	No	5 (36.8)	9 (64.3)

Heparin + means heparin did prescribed

Heparin _ means heparin did not prescribed

The rate of mortality with the history of VTE, anti-phospholipid syndrome and inherited thrombophilia was one in each group and all 3 received heparin as prophylaxis. There were 3 cases with sepsis and none of them got heparin.

In Table 4 we broke down the VTE events including timing and VTE score in each case.

**Table 4:** Timing and numbers of VTE score in each case

Cases	Time of VTE (per day after cesarean)	VTE score*
**1**	1	**5**
**2**	1	**7**
**3**	1	**5**
**4**	3	**5**
**5**	1	**6**
**6**	1	**1**
**7**	1	**8**
**8**	1	**5**
**9**	1	**8**
**10**	1	**4**
**11**	1	**5**
**12**	1	**3**
**13**	1	**3**
**14**	1	**7**
**15**	1	**6**
**16**	1	**7**
**17**	1	**3**
**18**	1	**2**
**19**	2	**4**
**20**	1	**6**
**21**	7	**5**
**22**	12	**9**
**23**	1	**4**
**24**	1	**2**
**25**	4	**3**
**26**	1	**1**
**27**	1	**2**

*VTE score based on ACOG criteria

We also compared the variables between the cases and a healthy control group and found out some variables including sepsis (*p* = 0.020), preterm labor (*p* = 0.002), stillbirth (*p* = 0.001), postpartum hemorrhage (*p* = 0.005), number of delivery (3 or more) (*P* = 0.016), blood transfusion (*P* = 0.003) and cardiac disease (*P* = 0.00) were significantly more in case group. Other variables were not significantly different between groups.

## Discussion

In this study, the risk factors for thromboembolism were investigated in 27 mortality cases due to mentioned reason. The mean gestational age was 33 weeks+ 6 days and nearly half of the cases experienced premature births. This is the same reason as Simpson et al. study that reported delivery at gestational age < 36 as a risk factor for VTE [19].

The use of risk score calculator may effectively and safely determine the duration of heparin therapy [18]. Darguad et al. indicated that in patients with moderate risk of VTE (score = 3–5), prescription of heparin prophylaxis in the third trimester prevent thrombosis [20]. In our study, the mean score of the thromboembolism (retrograde calculation) was 4.6 suggesting that these cases were a high-risk population for thromboembolic events; however only half of them with score more than 4 cases received heparin. There were some deliveries in deprived areas with no access to score calculation; therefore the Ministry of Health designed a single form to add the clinic and hospital records of each pregnant woman. Every center is obligated to calculate this score to decrease adverse events. This score will reevaluate in prenatal visit, delivery time and postpartum period.

Although the age of 35 years in pregnant women is associated with an increased risk of thromboembolic events [18], in this study the average age of the cases was 33.2 years that may reveal the impact of other variables to increase the risk of thromboembolism. Although Danilenko-Dixon et al. also reported no correlation between age and VTE in pregnancy and puerperium period [21].

More than 70% of cases have embolized in first day after delivery and about one half of them deceases intrapartum. Since more than half of them did not even receive a single dose of heparin, the need to quickly and accurately identify the indications of prophylactic treatment with heparin or other preventive methods to reduce mortality in these mothers is obvious.

Available data suggests that the risk of VTE is higher after cesarean section than vaginal delivery [22]. The indications for cesarean and the conditions during/after the surgery could be involved in the occurrence of thromboembolism and possibly mortality. The puerperium is the time of maximal risk of pregnancy-associated VTE and several observational studies have assessed the risk of VTE after cesarean section [23–25].

Blondon et al. accounted maternal Body mass index (BMI) as an important risk factor for postpartum VTE, grading from weak in overweight women to very strong in women with class III obesity [26]. However, in the other study, BMI was not related to VTE. This is similar to our result that BMI was in few patients above or equal to 30 and in none of the mortalities more than 40.

Women with inherited thrombophilia or a positive family history even with no previous episode of VTE, have a risk of developing a first VTE episode in pregnancy or postpartum period [27]. The highest risks were associated with homozygosity for factor V Leiden or the prothrombin G20210A variant [28]. In the present survey, one case had a history of VTE, one with an anti-phospholipid antibody syndrome, and the other with hereditary thrombophilia that all received heparin. Thromboembolism happened may be due to not receiving proper dosage of heparin.

Low molecular heparin is the preferred option for prophylaxis in most patients because of its better bioavailability, longer plasma half-life, more predictable dose response, and improved maternal safety [29, 30]. Also heparin is more effective than vitamin K antagonists in preventing recurrent VTE and post-thrombotic syndrome without increasing the risk of major bleeding events [31].

In our study, the most frequent blood group of mothers was A +. The study of Mirza Aghazadeh et al. also had the highest reports of VTE associated with the A + blood group [32]; Although this blood group generally has a high prevalence in Iran [33]. On the other population-based study blood groups A and AB were associated with increased risk estimates for VTE in pregnancy and the puerperium either [34].

Thirty-seven percent of mothers had more than 3 times delivery, 30% had heart disease, 21% had high blood pressure and 25% had stillbirth, it seems that these factors have a significant effect on the risk of maternal conditions.

Frequency of multiple pregnancy, severe bleeding, instrumental delivery, pregnancy by assisted reproductive techniques, prolonged delivery, sepsis, preeclampsia, and underlying illness were observed in less than 30% of these 27 mothers and showed that although these factors are predisposed to the occurrence of thrombotic events, but they do not contribute much to our study.

There are studies on the prevalence of risk factors and etiology of maternal mortality that explain rupture of membrane and severe bleeding are important risk factors in thromboembolism; therefore, precise follow-up and, if necessary, preventive and interventional proceedings may be effective in preventing maternal deaths.

## Conclusion

The study concludes that a high proportion of maternal mortality following thromboembolism are potentially preventable. It is suggested that pregnant women should be carefully evaluated for these risk factors, and that health care personnel and pregnant mothers should be trained to diagnose the early symptoms, as well as prevention and treatment of thromboembolic events.

The use of more accurate diagnostic methods for rapid diagnosis and control of the disease, as well as postpartum care, particularly in high-risk individuals, including women over 35 years of age, a history of more than three times pregnancy, a history of VTE, and the administration of prophylactic heparin with a sufficient dose and duration, decrease maternal mortality.

## Data Availability

The datasets used during the current study are available from the corresponding author on reasonable request. They are divided in two group. The data of the patients that declared in the article and are available with more detail by corresponding author and can be sent by her.

## Abbreviations

VTE: Venous Thromboembolism
BMI: Body Mass Index
NVD: Normal Vaginal Delivery
CS: Cesarean Section
IUFD: Intra Uterine Fetal Demise
IVF/ART: In Vitro Fertilization/ Assisted Reproductive Technology
SD: Standard Deviation
